# Optical Coherence Tomography Findings of Retinal Folds in Nanophthalmos

**DOI:** 10.1155/2011/491894

**Published:** 2012-01-23

**Authors:** F. Nilüfer Yalçındağ, Huban Atilla, Figen Batıoğlu

**Affiliations:** Department of Ophthalmology, Faculty of Medicine, Ankara University, 06100 Ankara, Turkey

## Abstract

*Aim*. To report the optical coherence tomography (OCT) findings in three members of the same family with nanophthalmos associated with elevated papillomacular retinal fold. *Methods*. Complete ophthalmic examination as well as ultrasonography and OCT was performed in all patients. *Results*. Axial lengths ranged from 16.75 mm to 17.48 mm and refractive errors ranged from +17.50 D to +20.50 D. Main fundus findings were the hyperopic crowded, cupless optic disc, and retinal fold through papillomacular region. Macular OCT scans revealed retinal fold with normal retinal pigment epithelium and choriocapillaris. *Interpretation*. It is presumed that the retinal folds in nanophthalmos result from a redundancy of the retinal layer caused by retarded growth of the scleral, choroidal, and retinal pigment epithelial layers. The anatomic information provided by the current study is consistent with this thesis.

## 1. Introduction

Nanophthalmos is a form of microphthalmia in which the anterior and posterior segments are abnormally small, but the lens has more normal dimensions, creating a high lens/globe volume ratio [1]. Nanophthalmic eyes typically exhibit high hyperopia. A wide variety of posterior segment changes, such as crowded optic disc and papillomacular fold, may be encountered in patients with nanophthalmos [2]. 

Optical coherence tomography (OCT) is a noninvasive diagnostic tool in evaluating fundus and especially macular diseases [3]. We report OCT findings in 2 siblings and their father with nanophthalmos associated with elevated papillomacular retinal fold.

## 2. Case Reports

### 2.1. Case  1

A 47-year-old male had axial lengths of 18.12 mm in the right eye (OD), 17.44 mm in the left eye (OS), and horizontal corneal diameters of 11 mm in both eyes (OU). His refraction was +17.50 D OU with cycloplegic refraction and best corrected visual acuity (BCVA) was 20/100 OD, OS. His biomicroscopy was normal with an intraocular pressure of 18 mmHg bilaterally. He had 30 prism diopters (PDs) of alternating esotropia with his full-corrected eyeglasses and torsional nystagmus. Fundus examination revealed bilateral crowded optic disc and elevated papillomacular fold. Fluorescein angiography (FA) showed a mild hypofluorescence in the fold area in both eyes. OCT demonstrated papillomacular fold of sensorial retina. B-scan ultrasonography showed sclerochoroidal thickening.

### 2.2. Case  2

**Figure 1:**
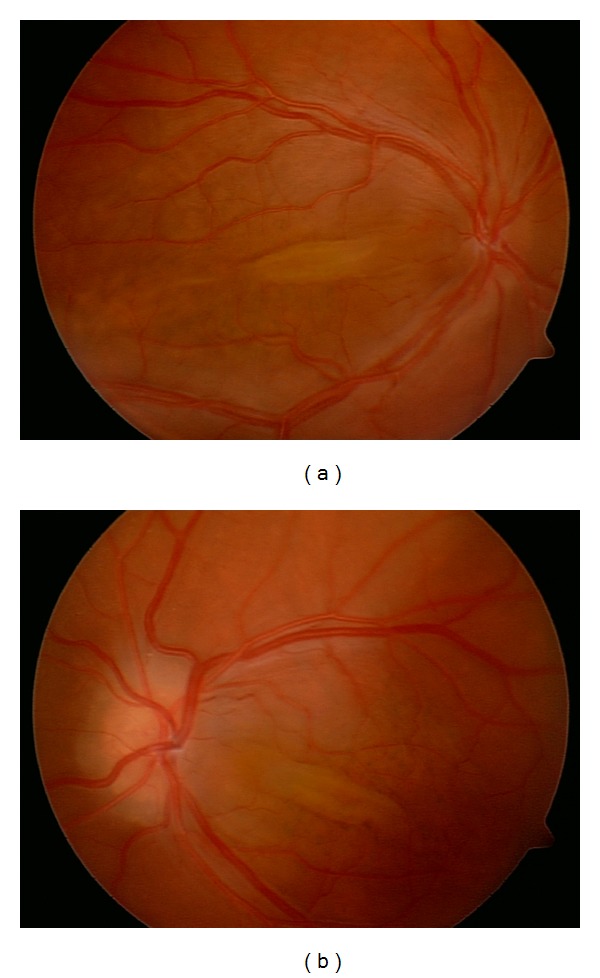
Fundus photographs of case 2 show elevated papillomacular retinal fold and crowded optic disc ((a): right, (b): left).

**Figure 2:**
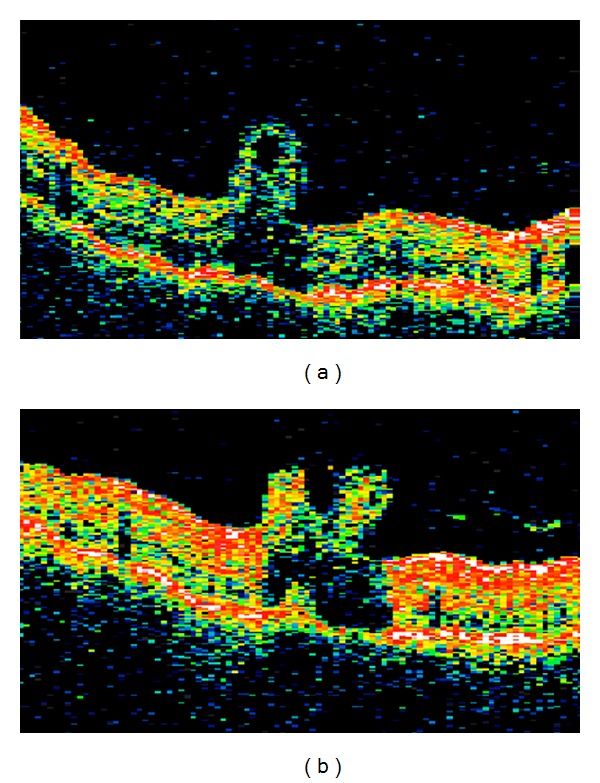
Vertical macular OCT scans of case 2 show neurosensory folding with subjacent optical shadowing. Note the uninvolvement of the retinal pigment epithelium in the fold area ((a): right, (b): left).

**Figure 3:**
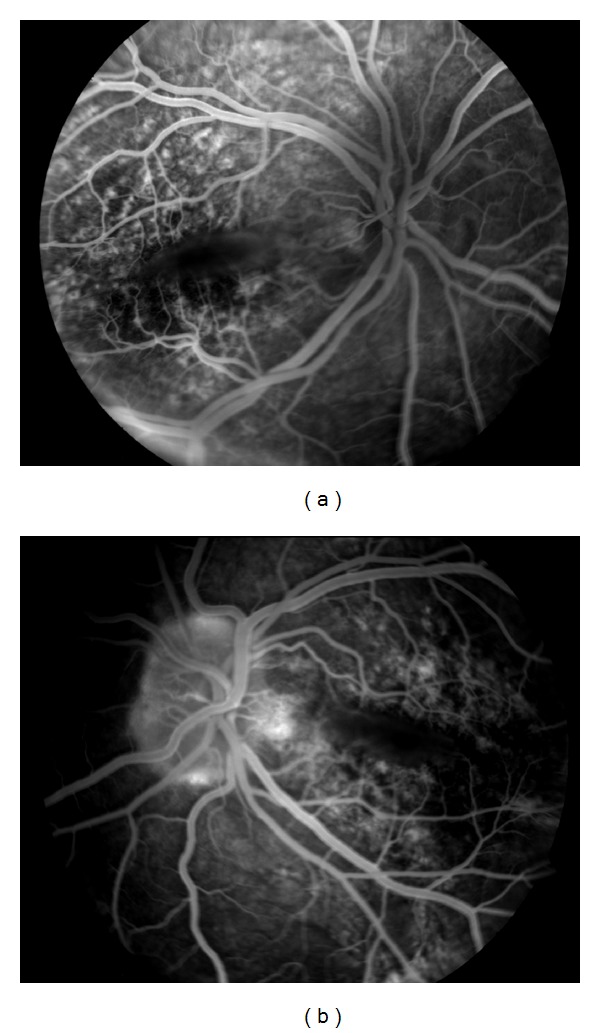
Fluorescein angiographies of case 2 show mottled hyperfluorescence due to pigment epithelial changes and mild hypofluorescence in the fold area ((a): right, (b): left).

Our second case was a 19-year-old male and was the son of case 1. He had axial lengths of 17.48 mm OD, 17.15 mm OS, and horizontal corneal diameters of 10 mm OU. His refraction was +20.50 D OU and BCVA was 20/100 OD and 20/200 OS. He was orthophoric with alternating cover test, but had a latent nystagmus. The intraocular pressure was measured as 20 mmHg OD and 19 mmHg OS. In fundoscopy, crowded optic disc, retinal pigment epithelial changes, and papillomacular retinal fold were detected in both eyes (Figures 1(a) and 1(b)). OCT revealed fold of sensorial retina (Figures 2(a) and 2(b)) and B-scan ultrasound images showed thickening of the sclera and choroid in both eyes. FA showed mottled hyperfluorescence due to pigment epithelial changes and mild hypofluorescence in the fold area bilaterally (Figures 3(a) and 3(b)).

### 2.3. Case  3

Our third case was a 16-year-old female and was the daughter of Case 1. She had axial lengths of 17.36 mm OD, 16.75 mm OS, and horizontal corneal diameters of 11 mm OU. Her cycloplegic refraction was +18.50 D OU and BCVA was 20/200 OD, OS. Motility examination revealed a 25-PD esotropia with full correction and she had a latent nystagmus similar to her brother. The intraocular pressure was measured as 16 mmHg OD, 18 mmHg OS. The posterior segment examination revealed bilateral crowded optic disc and elevated papillomacular fold. FA demonstrated a hypofluorescence in the fold area. OCT showed neurosensory folding OU. B-scan ultrasonography revealed diffuse sclerochoroidal thickening in both eyes.

## 3. Discussion

Optical coherence tomography demonstrated papillomacular retinal folds that were confined to the neurosensory retina with normal retina pigment epithelium and choriocapillaris in all of our patients. Timoney et al. reported chorioretinal folds involving both the retina and choroid by OCT in two cases of nanophthalmos associated with Kenny-Caffey syndrome [4]. Even though nanophthalmos generally is not associated with systemic abnormalities, Hallermann-Streiff-Francois syndrome, oculodentodigital syndrome, and Kenny-Caffey syndrome are three syndromes that need to be considered in patients with nanophthalmos [2, 5]. There were not any accompanying systemic findings suggesting any syndrome in our patients. Nanophthalmos may be inherited in sporadic, autosomal dominant, or autosomal recessive fashions. It seems to be inherited in an autosomal dominant manner in this family.

The term posterior microphthalmos describes eyes with microphthalmos that disproportionately affect the posterior ocular segment with normal external appearance of the eyes. Papillomacular folds are common among patients with posterior microphthalmos [6]. The OCT scans of patients with posterior microphthalmos and retinal folds have demonstrated that the neurosensory retina was folded, however the retinal pigment epithelium layer and choroid were intact without folding [7, 8]. Our findings are consistent with previous reports of OCT findings in posterior microphthalmos. In this aspect, we can assume that papillomacular folds have the same underlying mechanism in nanophthalmos and posterior microphthalmos that can be accepted as different manifestations of microphthalmos. On the other hand, Timoney et al. [4] showed that both choroid and retina were folded in their patients with nanophthalmos associated with Kenny-Caffey syndrome and this is not consistent with our findings. Involvement of underlying retinal pigment epithelium and choroid might be caused by other possible mechanisms due to accompanying systemic findings in Kenny-Caffey syndrome. 

It is presumed that the retinal folds result from a redundancy of the retinal layer caused by retarded growth of the scleral and retinal pigment epithelium layers [2, 9]. Scleral tissue examination in eyes with nanophthalmos has shown both thickening and less-ordered organization of collagen fibrils [10]. The thickened sclera does not impede the growth of the neurosensory retina, but influences the development of the choroid and the retinal pigment epithelium [9]. The anatomic information provided by the current study is consistent with this hypothesis.
